# Assessment of Platelet Function in Traumatic Brain Injury—A Retrospective Observational Study in the Neuro-Critical Care Setting

**DOI:** 10.3389/fneur.2018.00015

**Published:** 2018-01-26

**Authors:** Caroline Lindblad, Eric Peter Thelin, Michael Nekludov, Arvid Frostell, David W. Nelson, Mikael Svensson, Bo-Michael Bellander

**Affiliations:** ^1^Department of Clinical Neuroscience, Karolinska Institutet, Stockholm, Sweden; ^2^Division of Neurosurgery, Department of Clinical Neurosciences, University of Cambridge, Cambridge Biomedical Campus, Cambridge, United Kingdom; ^3^Department of Physiology and Pharmacology, Section of Perioperative Medicine and Intensive Care, Karolinska Institutet, Stockholm, Sweden

**Keywords:** traumatic brain injury, progressive hemorrhagic injury, platelet aggregation, platelet aggregation inhibitors, multiple electrode aggregometry, cyclooxygenase inhibitors

## Abstract

**Background:**

Despite seemingly functional coagulation, hemorrhagic lesion progression is a common and devastating condition following traumatic brain injury (TBI), stressing the need for new diagnostic techniques. Multiple electrode aggregometry (MEA) measures platelet function and could aid in coagulopathy assessment following TBI. The aims of this study were to evaluate MEA temporal dynamics, influence of concomitant therapy, and its capabilities to predict lesion progression and clinical outcome in a TBI cohort.

**Material and methods:**

Adult TBI patients in a neurointensive care unit that underwent MEA sampling were retrospectively included. MEA was sampled if the patient was treated with antiplatelet therapy, bled heavily during surgery, or had abnormal baseline coagulation values. We assessed platelet activation pathways involving the arachidonic acid receptor (ASPI), P2Y_12_ receptor, and thrombin receptor (TRAP). ASPI was the primary focus of analysis. If several samples were obtained, they were included. Retrospective data were extracted from hospital charts. Outcome variables were radiologic hemorrhagic progression and Glasgow Outcome Scale assessed prospectively at 12 months posttrauma. MEA levels were compared between patients on antiplatelet therapy. Linear mixed effect models and uni-/multivariable regression models were used to study longitudinal dynamics, hemorrhagic progression and outcome, respectively.

**Results:**

In total, 178 patients were included (48% unfavorable outcome). ASPI levels increased from initially low values in a time-dependent fashion (*p* < 0.001). Patients on cyclooxygenase inhibitors demonstrated low ASPI levels (*p* < 0.001), while platelet transfusion increased them (*p* < 0.001). The first ASPI (*p* = 0.039) and TRAP (*p* = 0.009) were significant predictors of outcome, but not lesion progression, in univariate analyses. In multivariable analysis, MEA values were not independently correlated with outcome.

**Conclusion:**

A general longitudinal trend of MEA is identified in this TBI cohort, even in patients without known antiplatelet therapies. Values appear also affected by platelet inhibitory treatment and by platelet transfusions. While significant in univariate models to predict outcome, MEA values did not independently correlate to outcome or lesion progression in multivariable analyses. Further prospective studies to monitor coagulation in TBI patients are warranted, in particular the interpretation of pathological MEA values in patients without antiplatelet therapies.

## Introduction

Traumatic brain injury (TBI) is a worldwide leading cause of mortality and disability (1). Following the acute phase, TBI is characterized by the development of secondary injuries (1), of which one is trauma-induced coagulopathy (TIC) (2). In severe TBI, the incidence of coagulopathy exceeds 60% (2) and is associated with lesion progression, mortality (3), and poor outcome (2). Alarmingly, despite conventional coagulation parameters within normal reference intervals, radiologic progression of intracranial lesions still occurs among more than 30% of patients (4). Moreover, the TBI setting now comprises increasingly older patients (5) for which antiplatelet therapy is common (6, 7), but associated with lesion progression and unfavorable outcome (7, 8). Consequently, hemorrhagic progression is the leading cause of seemingly preventable death (9) following TBI. In aggregate, there is considerable interest in diagnosis and treatment of coagulopathy in TBI patients, especially in the case of anticoagulative therapies.

Diagnosis of coagulopathy is complex (2, 10). Routine platelet and coagulation tests comprising platelet count, activated partial thromboplastin time (APTT), and international normalized ratio (2, 11) have been shown to be insufficient to diagnose the full perspective of coagulopathies (4). Similarly, more advanced laboratory tests, including thromboelastography and rotational thromboelastometry, do not adequately predict coagulopathy (10). Of the aforementioned, platelet count is commonly used to assess primary hemostasis and risk for bleeding progression, but platelet count is a quantitative measurement that does not account for platelet functionality (11). This has led to the development of platelet function assessment techniques (12). Among the methods described in Ref. (12), Multiplate^®^ (Roche Diagnostics, Basel, Switzerland) and VerifyNow^®^ (Accumetrics, San Diego, CA, USA) correlate with light transmittance aggregometry, the gold standard method for assessing platelet function (12).

While these techniques are primarily used to assess platelet function in cardiology patients, Multiplate^®^, based on multiple electrode aggregometry (MEA), use was shown to improve outcome prediction in a general trauma population (13). However, in TBI cohorts, the utility of MEA remains to be convincingly shown. In spite of this, MEA is used with increasing frequency in clinical practice, demonstrating the clinicians’ need to evaluate platelet function and improve coagulation assessment within the field of TBI.

In our department, a majority of patients with TBI in need of neurointensive care unit (NICU) care is assessed using MEA. This presents a unique opportunity to evaluate its utility within the clinical setting. With this in mind, we sought to characterize MEA alterations following TBI, analyze how MEA levels are altered in drug induced coagulopathy, examine how MEA levels are associated with bleeding progression in patients with severe TBI, and how it subsequentially affects outcome.

## Materials and Methods

This was a retrospective observational study undertaken at the NICU at Karolinska University Hospital (Stockholm, Sweden) including patients treated between February 2010 (clinical introduction of MEA) and May 2014. The work was approved by the local ethics committee in Stockholm County, the Central Ethical Review Board (diary numbers 2014/1488-31/5 and 2015/1675-31/1).

### Study Criteria

Patients were included if they had suffered a traumatic intracranial lesion, were of ≥15 years of age, and had been admitted to the NICU during the years 2010–2014. All patients included were at all times treated according to local routine at the NICU, as earlier described in Ref. (14).

### Definitions

Trauma severity was assessed using the definitions of the Advanced Trauma and Life Support system (15) and is described as Injury Severity Score (ISS) (16). CT scans were evaluated by a trained examiner, blinded from coagulation measurements and outcome. Radiological parameters included midline shift (in mm), traumatic subarachnoid hemorrhage, epidural hematoma, acute subdural hematoma, and cerebral contusions. CT scans were graded according to Ref. (17), Rotterdam CT score (18), and Stockholm CT score (19). Stockholm CT score was used in the models as it has been shown to be the more accurate outcome predictor of these (20). The admission and follow-up CT scans were evaluated with regard to radiologic intracranial hemorrhagic progression. All types of intra- and extra-parenchymal lesions on the follow-up brain CT scan were compared with the initial scan and if any progression had occurred, it was noted (14). If the patient underwent surgical evacuation of an intracranial lesion, any un-evacuated hematomas present were assessed for progression. Data including Glasgow Coma Scale (GCS), ISS, and outcomes were collected prospectively in the Karolinska Traumatic Injury database. Additional parameters were collected retrospectively through the electronic medical record system TakeCare^®^ (CompuGroup Medical Sweden AB, Farsta, Sweden), and any missing was be interpreted as not performed as there is no loss of data within the system.

### Outcome

Glasgow Outcome Scale (GOS) (21) was evaluated at approximately 12 months following the trauma by questionnaire concerning functional outcome. GOS is classified as a categorical variable, where GOS 1 indicates death, GOS 2 vegetative state, GOS 3 severe dependent state, GOS 4 moderate independent state, and GOS 5 full recovery. Dichotomized GOS comprises the two categories GOS 1–3 (unfavorable) and GOS 4–5 (favorable) outcome.

### MEA Measurements

The Multiplate^®^ (Roche Diagnostics, Basel, Switzerland) unit was used to assess MEA values. This is an impedance aggregometry method (22) where multiple electrodes are immersed in a blood sample. Upon initial contact with blood, platelets coat the electrodes. Following stimulation with platelet agonists, there is a large increment in platelet aggregation upon the electrodes, quantified as increased impedance (23), and measured as an area under the curve (AU), providing the measurement unit. The method has been previously used in settings comprising intracranial pathology (24).

Using MEA, we evaluated three different platelet activation pathways: the arachidonic acid receptor (ASPI), P2Y_12_ receptor (ADP), and thrombin receptor (TRAP). These receptors are specifically inhibited by the pharmacological compounds: cyclooxygenase (COX) inhibitors (ASPI), P2Y_12_ inhibitors (ADP), and glycoprotein IIb/IIIa antagonists (TRAP) (23). Indications for MEA tests were as follows: antiplatelet therapy; heavy perioperative bleeding judged by the surgeon and/or intensivist; clinical bleeding propensity; or abnormal baseline hematologic values. MEA tests were occasionally taken solely at the initiative of a clinician. Commonly, if a first sample was obtained and particularly, if it guided treatment, the initial measurement was followed by subsequent measurements to evaluate treatment effect. Currently, there are no internationally established guidelines advising when MEA samples should be acquired in NICU TBI patients. In this study, up to nine MEA measurements were recorded per patient. The “first value” was defined as the value in closest proximity to admission. Two different MEA devices (both Multiplate^®^) were used. The reference intervals were similar for the two devices, with the exception of ASPI, which was 71–115 AU in a local NICU apparatus and 65–119 AU in the central laboratory apparatus. The reference interval for the local apparatus was used in further analyses.

### Statistical Analysis

The full statistical protocol is described in the Supplementary Materials and Methods in the Supplementary Material. Demographic data were presented as mean ± SD, median (interquartile range), or count (%). Spearman correlation (rho, ρ) was used to assess correlation between different platelet receptor values, in addition to visual scatterplots. The distribution of MEA was examined using the Shapiro–Wilk test, and inferential analysis was conducted using the Mann–Whitney *U* test and the Wilcoxon signed rank test where appropriate. We assessed longitudinal changes of MEA values (ASPI) using linear mixed effect models with random and fixed effects in the lme4 package (25) in R. Model criteria were evaluated graphically and deemed to be fulfilled. The compiled model was obtained using likelihood ratio tests, where after the model with the lowest Akaike Information Criterion value was chosen. Various outcome prediction models were used. First, to assist hypothesis generation, outcome was visualized using recursive partitioning decision trees in the R package rpart (26) and rattle (27) with GOS as dependent variable (21). Independent variables were those previously shown to be the major predictors for TBI outcome [International Mission for Prognosis and Analysis of Clinical Trials in TBI (IMPACT) variables] (28), combined with coagulation tests clinically available at our clinic, including the first MEA values. Regression models were used to assess association with outcome (GOS, proportional odds) and hemorrhagic progression (logistical). The univariate analysis on outcome was performed with unimputed data. Variables included in univariate analysis were chosen with guidance from Fabbri and colleagues (7), or if hypothesized to modulate platelet function or coagulation. Variables emanating significant in univariate analysis were analyzed in a step-up followed by a step-down model, ultimately resulting in the final multivariable model using the R package rms (29). The statistical software program Rstudio^®^ (R Foundation for Statistical Computing, Vienna, Austria; http://www.R-project.org) was used in all calculations.

### Missing Data

Missing values relevant for outcome analysis were plotted (Figure S1 in Supplementary Material) using the R package neato (30). As several variables contained missing values to a large extent, we employed a standard multiple imputation approach using seven imputations of the dataset using the R package mice (31). The individual imputations and the pooled result from them were used in subsequent analyses. This approach has been used by the IMPACT study group (32) and is favored by the statistical literature (33).

## Results

### Demographics

Between 2010 and 2014, 387 TBI patients were admitted to the NICU at the Karolinska University Hospital. Of these, 178 underwent MEA analysis and were included. Patient demographics are presented in Tables 1 and 2, respectively. Admission GCS varied from 3 to 15, with a median GCS of 7 (severe TBI). In total, 48% suffered an unfavorable outcome. At hospital admission, 24% of the patients had COX inhibitor treatment. The median MEA values for the first ASPI and ADP measurements were lower than the reference interval, meanwhile the first TRAP measurement was within it. Of all patients’ first MEA values, 124 (70%) had pathologically low ASPI values, 109 patients (61%) had low ADP, and 65 patients (37%) low TRAP values. Throughout the hospital stay, 112 patients (63%) received a platelet transfusion, with a median total volume of 600 ml.

**Table 1:** Patient demographics.

Variable	Type (unit)	Total number of patients: 178
Gender	Male/female	133/45 (75/25)
Age	(years)	54 (37–65)
Oxygen saturation SoA	(%)	96 (93–98)
	Missing	68 (38)
Blood pressure SoA	Systolic (mmHg)	130 (120–150)
	Missing	69 (39)
Admission GCS	GCS 3	53 (30)
	GCS 4–5	15 (8)
	GCS 6–8	31 (17)
	GCS 9–13	47 (26)
	GCS 14–15	32 (18)
	Median	7
Pupil responsiveness admission	Normal	138 (78)
	Unilateral unresponsive	18 (10)
	Bilateral unresponsive	19 (11)
	Missing	3 (1.7)
Extracranial injury (multitrauma)	Present	46 (26)
Radiological findings	Midline shift (mm)	3 (0–9)
	Progression hematoma	60 (34)
	Epidural hematoma	20 (11)
	Dual subdural hematoma	11 (6)
	Intraventricular bleeding	28 (16)
	Subarachnoid hemorrhage basal cisterns	42 (24)
	Subarachnoid hemorrhage convexity	121 (68)
Stockholm CT score	Total score	22 ± 9.6
Final GOS	GOS 1 (dead)	22 (12)
	GOS 2 (vegetative)	2 (1)
	GOS 3 (severe, dependent)	61 (34)
	GOS 4 (moderate, independent)	56 (31)
	GOS 5 (recovered)	37 (21)
	GOS 1–3 (unfavorable)	85 (48)
	GOS 4–5 (favorable)	93 (52)

*In total, 178 patients were included. Data are depicted as count (%) if categorical and otherwise as median (interquartile range) or mean ± SD, if continuous. If there were any missing values, they are denoted as count (%) after each variable of concern*.

*GCS, Glasgow Coma Scale; GOS, Glasgow Outcome Scale; SoA, scene of accident*.

**Table 2:** Coagulation status.

Variable		Patient values	Reference interval (unit)
Platelet count admission		212 (175–248)	145–348 (10^9^/l)
APTT admission		31 (29–36)	28–40 (s)
	Missing	9 (5)	
INR admission		1.1 (1–1.2)	<1.2 (INR)
	Missing	6 (3)	
ASPI, first value		51 (23.5–79)	71–115; 65–119 (AU)
ADP, first value		51 (31–69)	57–113; 57–113 (AU)
TRAP, first value		95 ± 35.6	84–128; 84–128 (AU)
	Missing	1 (0.56)	
Platelet transfusion	Transfused	112 (63)	
	Missing	4 (2)	
Platelet transfusion dose	Volume	600 (0–1,142)	(ml)
	Missing	10 (5.6)	
Erythrocyte transfusion	Transfused	102 (57)	
Fresh frozen plasma transfusion	Transfused	80 (45)	
COX inhibitor treatment	Treatment before admission	42 (24)	
ADP inhibitor treatment	Treatment before admission	5 (2.8)	

*In total, 178 patients were included. Data are depicted as count (%) if categorical and otherwise as median (interquartile range), or mean ± SD, if continuous. When applicable, reference intervals are reported as those that at the time point of the study were used at the Karolinska University Hospital. Measurement units are given in parentheses next to the reference interval value. MEA measurements were obtained using two different devices, which had slightly different reference intervals, both of which are reported*.

*ADP, P2Y_12_ receptor; APTT, activated partial thromboplastin time; ASPI, arachidonic acid receptor; AU, area under the curve; COX, cyclooxygenase; INR, international normalized ratio; TRAP, thrombin receptor; MEA, multiple electrode aggregometry*.

### Correlation between Different MEA Variables

There was a significant positive correlation between all three platelet receptors’ first MEA values (Figures S2A1–C1 in Supplementary Material). For the ASPI versus TRAP receptor, ρ = 0.59 (*p* < 0.001). For the ASPI versus ADP receptor, ρ = 0.71 (*p* < 0.001). Finally, for the ADP versus the TRAP receptor, ρ = 0.66 (*p* < 0.001). Based on this, we pursued our examinations primarily using the ASPI receptor.

### Platelet Modulating Pharmacological Compounds

**Figure 1:**
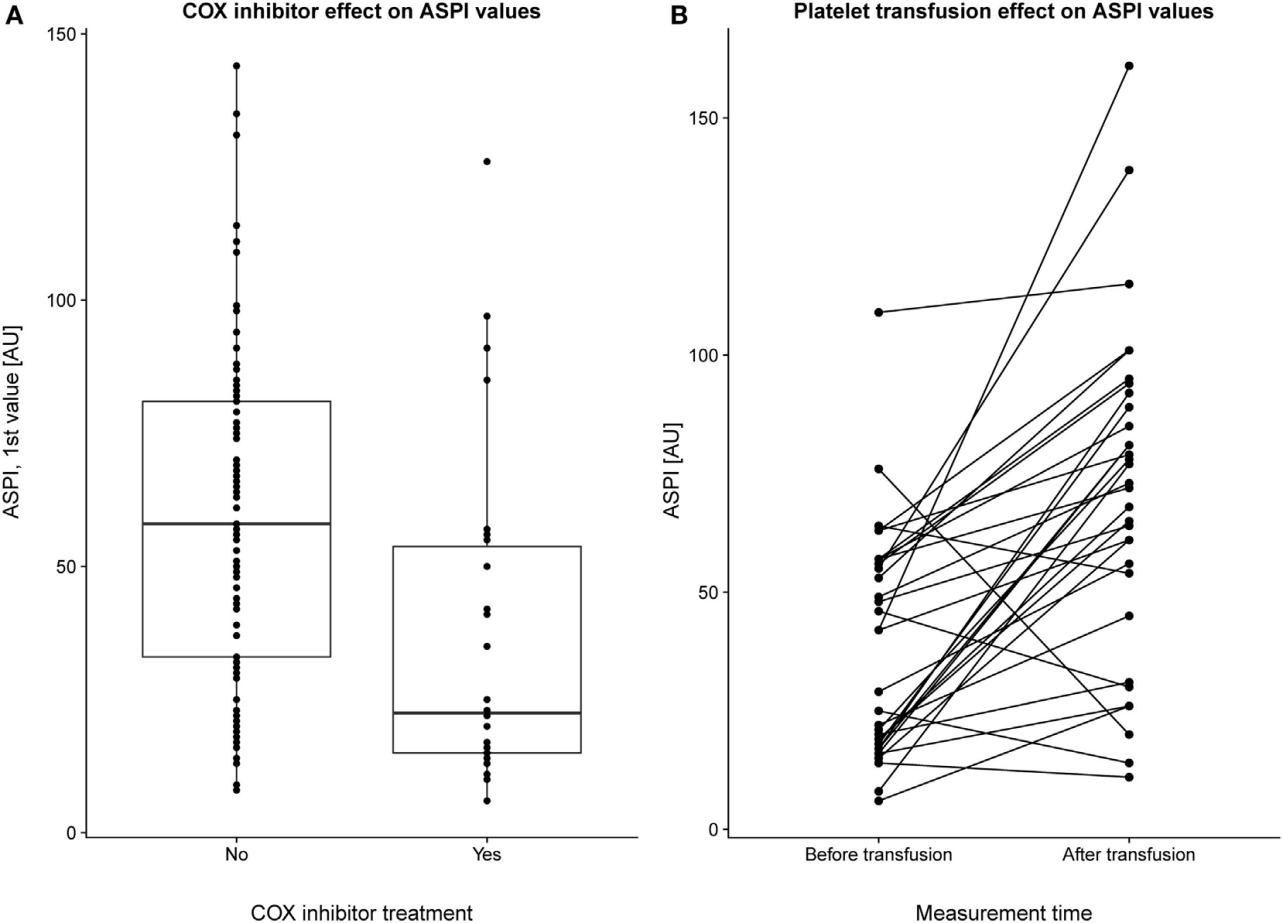
Pharmacologic modulation of multiple electrode aggregometry (MEA) values. The first arachidonic acid receptor (ASPI) values (indicated as individual data points and as summarizing boxplots) for a subset of patients with and without cyclooxygenase (COX) inhibitor treatment (and without preceding platelet transfusions) are depicted **(A)**. Overall, COX inhibitor treatment yielded lower ASPI results, *p* < 0.001. In panel **(B)**, a subgroup of patients who had undergone MEA measurements before and after transfusion were selected. Individuals are plotted with their respective ASPI value at the first and second measurement, with a line connecting each individual. No account was taken for how long time that had passed between the first and second MEA measurement, but there was still a strongly significant (*p* < 0.001) increase in platelet function following transfusion.

The first MEA values were examined on 127 patients of which 30 had received COX inhibitor treatment. We excluded all patients who had received a platelet transfusion before MEA values were obtained. MEA values were lower for patients treated with COX inhibitors compared with those who were not (Figure 1A), *p* < 0.001. Notably, the median ASPI value was below the lower reference interval, independent of COX inhibitor therapy. In total, ~66% of patients without COX inhibitor treatment demonstrated ASPI values below the lower reference interval. MEA values increased following a platelet transfusion (Figure 1B), *p* < 0.001.

### Longitudinal Pattern

**Figure 2:**
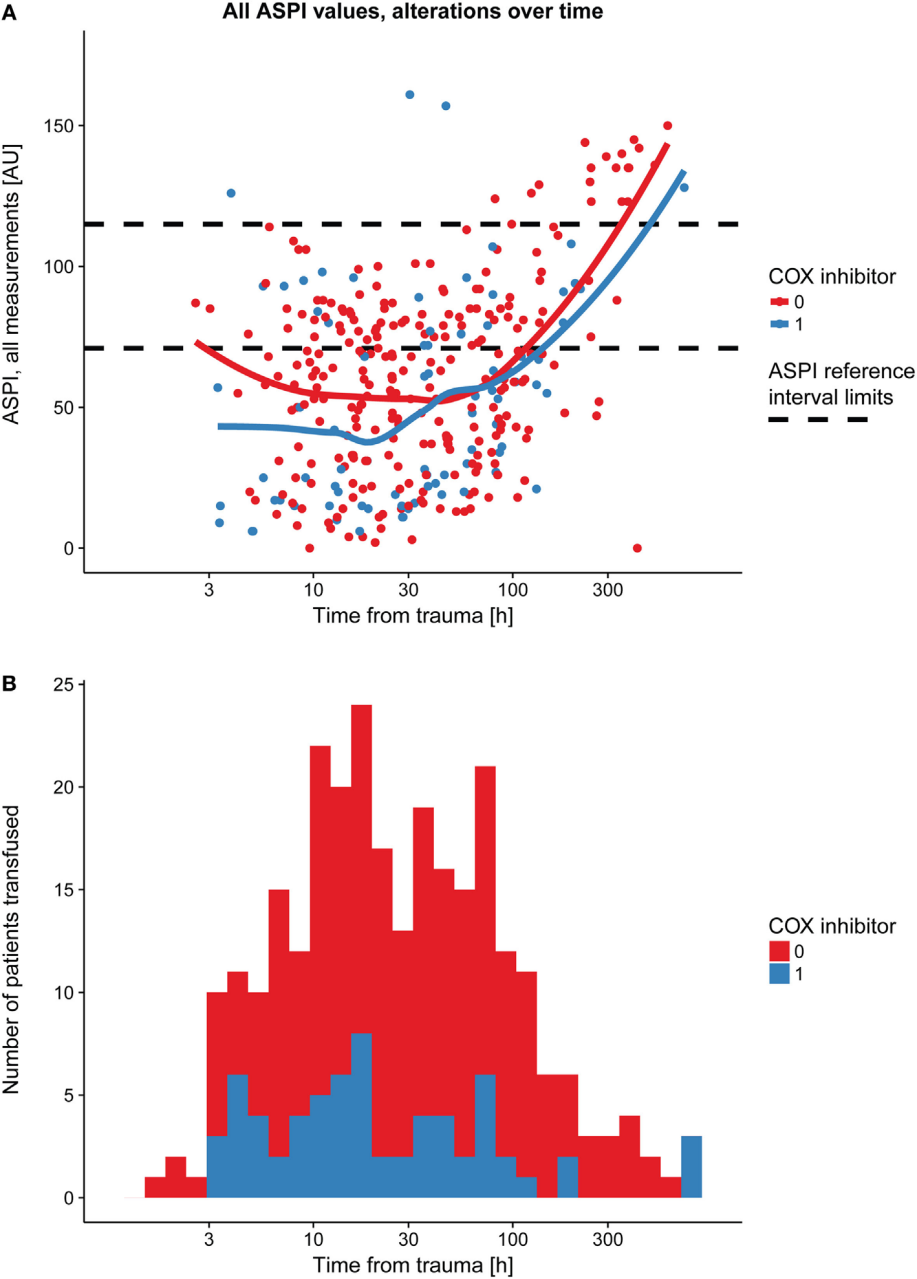
Platelet function alterations over time following TBI, dependent on prehospital cyclooxygenase (COX) inhibitor treatment. Patients with COX inhibitor treatment before hospital admission demonstrated a consistently lower arachidonic acid receptor (ASPI) value compared with those without COX inhibitor treatment **(A)**, as showed by the two LOWESS curves. The black dashed line denotes the ASPI reference interval (71–115 AU). At one time point, however, there was a partial overlap, which, as shown in panel **(B)**, might be explained by an increase in platelet transfusions at the time point preceding the overlapping LOWESS curves in panel **(A)**. Abbreviations: AU, area under the curve; LOWESS, locally weighted scatterplot smoother; TBI, traumatic brain injury.

Immediately following TBI, platelet function (ASPI) was low and thereafter increased over time (Figure 2A; Table 3, *p* < 0.001). The same graphical trend was seen when excluding patients on COX inhibitors (Figure S3B in Supplementary Material), or that had been transfused with platelets (Figure S3C in Supplementary Material). Moreover, platelet count was positively correlated with MEA values (Figure S4 in Supplementary Material; Table 3, *p* < 0.001). Patients presenting with ongoing COX inhibitor treatment had consistently lower ASPI values than patients not treated with COX inhibitors (Figure 2A; Table 3, *p* = 0.026), except for one time point, which, however, was preceded by an increase in the number of platelet transfusions (Figure 2B).

**Table 3:** Linear mixed effect model for ASPI values among TBI patients.

Fixed effect variable	Estimate	SE	*p*Value (likelihood ratio test)
Time from trauma	0.10627	0.02	<0.001
Platelet count	0.09221	0.01381	<0.001
COX inhibitor	−9.47869	4.23748	0.02583

*Random intercepts: *p* < 0.001 [*p* value generated using (34)]*.

*AIC value final model: 3,265.7*.

*Variables affecting ASPI values were compared across measurement time points. Variables that affected ASPI when examined graphically were included in the model. *p* Values were obtained using the likelihood ratio test, or as otherwise reported. Included random effects were random intercepts (subject ID), presented at the bottom of the table*.

*AIC, Akaike Information Criterion; ASPI, arachidonic acid receptor; COX, cyclooxygenase; TBI, traumatic brain injury*.

### MEA As Predictor of Hemorrhagic Progression and Outcome

#### Outcome Illustration Using Decision Trees

**Figure 3:**
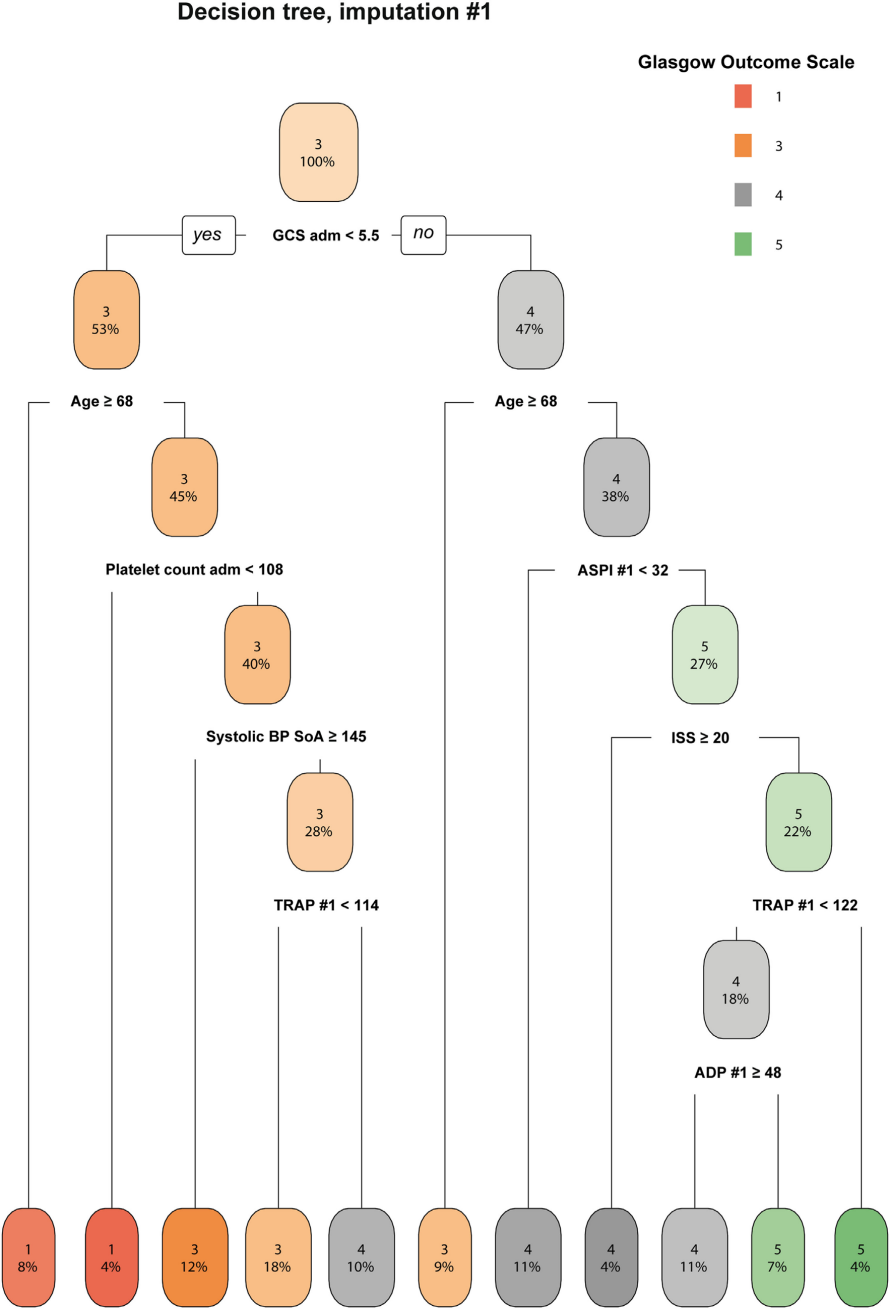
A decision tree for determining the importance of ASPI. A representative imputation of one of the decision trees is depicted. Each node in the tree denotes the predicted GOS value (indexed by color and GOS category number), followed by the percentage of patients belonging in each node. While moving downwards in the tree, GOS changes depending on the independent variables and the percentages of patients who pertain to the stipulated criteria decrease accordingly. Consistent across all imputations, ASPI values in the range of 32–68 affected the determination of Glasgow Outcome Scale (GOS) 4–5 among younger patients with higher GCS (only one imputation shown). Radiologic score was defined as the Stockholm CT score (19). Data values are presented in the same units as can be found in Tables 1 and 2, respectively. Abbreviations: adm, admission; ADP, P2Y_12_ receptor; ASPI, arachidonic acid receptor; BP, blood pressure; GCS, Glasgow Coma Scale; ISS, Injury Severity Score; SoA, scene of accident; TRAP, thrombin receptor; #, ordered number of sample; GOS, Glasgow Outcome Scale.

The decision tree gives a graphic representation of information content that could aid rule based interpretation of the data. Decision trees from all seven imputations were compared visually and showed congruency overall (data not shown), of which one representative is shown (Figure 3). Low first ASPI values appear to distinguish worse outcome in patients <68 years with higher GCS (≥5.5) scores at admission. Although already on level three of the decision tree, this degree of subgrouping must be interpreted with care and as hypothesis generating.

#### Regression Analyses of MEA versus Outcome and Hemorrhagic Progression

**Figure 4:**
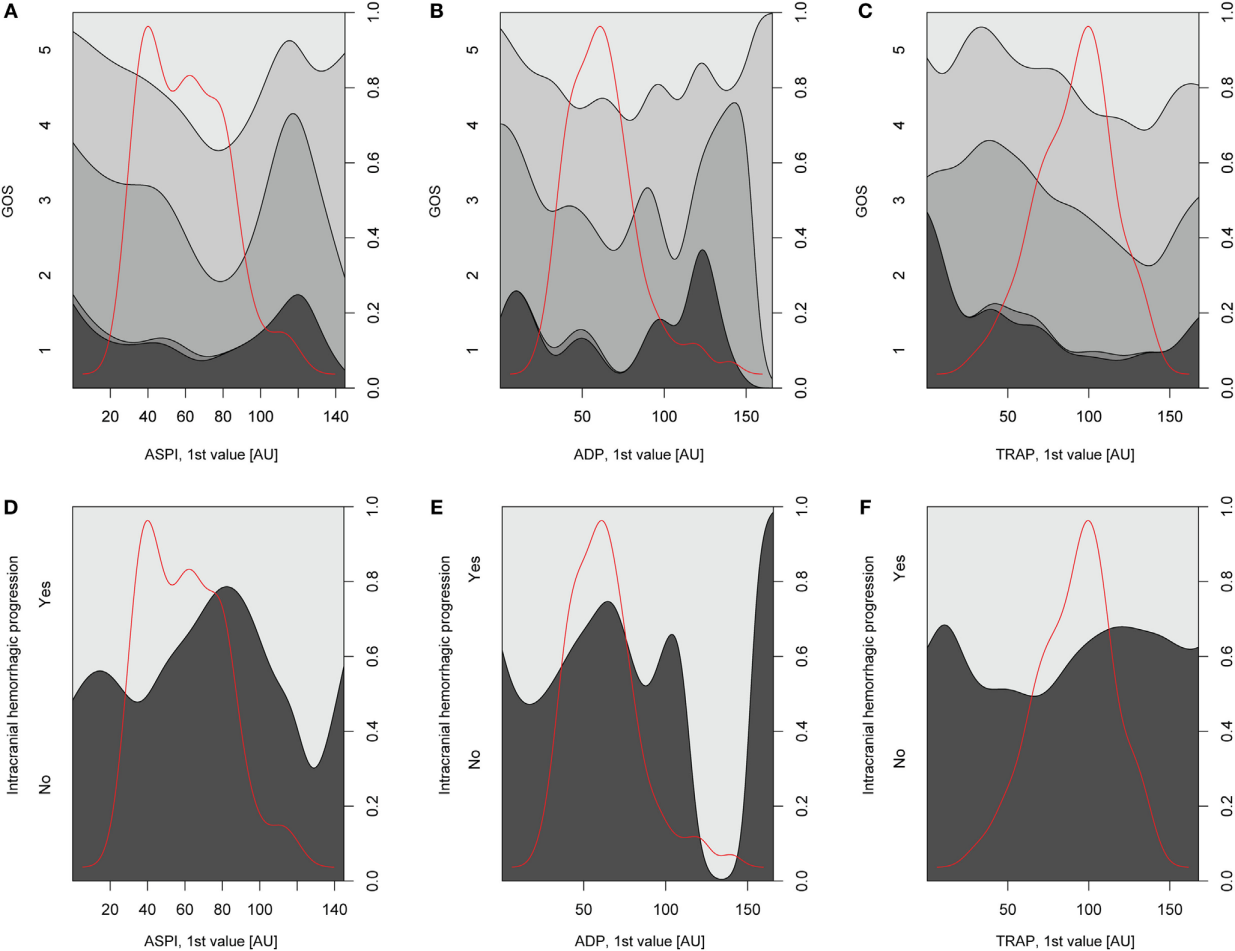
Depiction of univariate analysis of selected variables hypothesized to influence Glasgow Outcome Scale (GOS) or hemorrhagic progression. In panels **(A–C)**, conditional density (CD) plots for the different platelet receptors versus GOS are demonstrated. In a CD plot, the left *y*-axis depicts the category of the dependent variable (in this case GOS). The right *y*-axis depicts outcome proportions for each value of the *x*-axis’ independent variable (in this case the different platelet receptors). The red line overlaid in the plot depicts the amount of observations across different values of the independent variable and has therefore no relation with the *y*-axis. As the density plot demonstrates, there were fewer patients with extreme values of the independent variable, respectively, thus decreasing the reliability of the relationship between the independent variable and GOS at these values of the independent variable. In panel **(A)**, arachidonic acid receptor (ASPI) values generated high amounts of unfavorable outcome (GOS 1–3) at low and high ASPI values, respectively, although there was scarce amount of data for higher ASPI values. The same trend was not readily detectable for the P2Y_12_ receptor (ADP) due to a fluctuating curve **(B)** but was evident for the TRAP **(C)**. In panels **(D–F)**, CD plots for the different platelet receptors versus hemorrhagic progression are depicted. A similar u-shaped relation as in panels **(A–C)** was detected for ASPI but now toward increased risk of hemorrhagic progression **(D)**. A similar trend was seen in **(E)** but not as readily in panel **(F)**, depicting the P2Y_12_ (ADP) receptor and thrombin receptor (TRAP), respectively.

Relations of MEA values toward GOS levels were visualized with conditional density plots (CD plots, Figures 4A–C). ASPI values had a u-shaped relation to GOS (Figure 4A), where both high and low levels were associated with a more unfavorable outcome. Although areas with limited data must be interpreted with caution, the CD plots can help visualize the information content, and accuracy of univariate regression will be related to the slope of the lines discriminating levels in areas with most data. ADP showed a similar tendency as ASPI, but fluctuations probably reflecting “noise,” make interpretation difficult (Figure 4B). Higher TRAP values were visually associated with favorable GOS (Figure 4C). The results of the univariate analysis (Table 4) were congruent with the CD plots, with ASPI (*p* = 0.0389) and TRAP (*p* = 0.0086), but not ADP being significantly related to GOS. The IMPACT variables (28) that form the “base model” [age, admission GCS, pupil responsiveness, Stockholm CT score, oxygen saturation, and blood pressure (BP)] were significant in univariate analysis with the exception of BP. Additional significant variables in univariate analysis were the coagulation predictors APTT (*p* = 0.0020), COX inhibitor treatment (*p* = 0.0176), platelet transfusion (*p* = 0.0030), and intracranial radiologic hemorrhagic progression (*p* = 0.0001). The ISS was not significantly related to outcome. Following step-up (Table S1 in Supplementary Material) and step-down models, radiologic intracranial hemorrhagic progression was the only significant variable aside from the base model in the multivariable model (Table 5). Overall, the Nagelkerke’s pseudo-*R*^2^ had a mean of 39.8% of the pseudo- explained variance of outcome prediction, for the seven different imputations. Since elderly patients are more likely to have COX inhibitors, the age variable in IMPACT might affect the interpretation of COX inhibitors. Omitting age from the base model resulted in COX inhibitors being a significant predictor of worse outcome in the multivariable model (data not shown).

**Table 4:** Univariate analysis of variables and correlations to final GOS.

Independent variable	*p*Value	Pseudo-*R*^2^
**Base model**		
Age	0.0008	0.067
GCS at admission	<0.0001	0.167
Pupil responsiveness	0.0070, <0.0001	0.130
Stockholm CT score	<0.0001	0.208
Oxygen saturation at SoA	0.0282	0.044
Blood pressure at SoA	0.1401	NS
**Additional variables assessed**		
Injury Severity Score	0.4317	NS
ASPI, 1st value	0.0389	0.026
ADP, 1st value	0.2874	NS
TRAP, 1st value	0.0086	0.042
TPK at admission	0.3616	NS
INR at admission	0.4198	NS
APTT at admission	0.0020	0.060
Platelet transfusion	0.0030	0.054
COX inhibitor treatment	0.0176	0.034
Radiologic intracranial hemorrhagic progression	0.0001	0.104

*Results from univariate analysis of independent variables assumed to affect Glasgow Outcome Scale (GOS) are depicted. Apart from the IMPACT variables (28), these comprised platelet count, coagulation measurements, variables hypothesized to modulate platelet function, and radiologic intracranial hemorrhagic progression. Platelet transfusion, COX inhibitor treatment, and radiologic intracranial hemorrhagic progression were used as dichotomous variables*.

*ADP, P2Y_12_ receptor; APTT, activated partial thromboplastin time; ASPI, arachidonic acid receptor; COX, cyclooxygenase; CT, computerized tomography; GCS, Glasgow Coma Scale; INR, international normalized ratio; SoA, scene of accident; TRAP, thrombin receptor*.

**Table 5:** Multivariable proportional odds analysis of variables affecting final GOS.

Independent variable	OR	CI	*p*Value
Age	0.966	0.948–0.984	0.000117
GCS admission	1.130	1.05–1.21	0.001612
Pupil responsiveness	0.596	0.355–1.00	0.0707
Stockholm CT score	0.945	0.914–0.977	0.00176
Oxygen saturation SoA	1.06	1.00–1.11	0.0685
Blood pressure SoA	1.00	0.992–1.01	0.409
Radiological intracranial hemorrhagic progression	0.363	0.196–0.671	0.00479

Mean Nagelkerke’s pseudo-R^2^ 0.398 = 39.8%

*Results from multivariable analysis of independent variables assumed to affect Glasgow Outcome Scale (GOS) are depicted. Data are presented as odds ratio (OR) and 95% confidence interval (CI) for one imputation. *p* Values are reported as the pooled *p* value from all imputations (*n* = 7)*.

*CT, computerized tomography; GCS, Glasgow Coma Scale; SoA, scene of accident*.

Relations of MEA as predictors toward hemorrhagic progression were visualized with CD plots (Figures 4D–F) suggesting ASPI values to have a u-shaped association with an increased percentage of hemorrhagic progression (Figure 4D), similar to GOS. However, this was not significant in univariate analysis (Table S2 in Supplementary Material). APTT was significant in univariable analysis (*p* = 0.0178, Nagelkerke’s pseudo-*R*^2^ = 0.058, Table S2 in Supplementary Material). Admission GCS and Stockholm CT score were borderline significant in univariate analysis (Table S2 in Supplementary Material). Since no variables were significant except APTT, no step-down or multivariable analysis was conducted.

## Discussion

To the best of our knowledge, this is the first study to investigate the clinical utility of a platelet function method in a clinical TBI setting. We found that

(i) Platelet function, as indicated by MEA, exhibits a temporal profile even in the absence of platelet inhibitors where MEA values are generally low initially, and subsequently increase over the days following TBI. If this also reflects a clinically significant coagulopathy is yet unknown.
(ii) The first ASPI and TRAP values were associated with long-term outcome in univariable analyses, albeit they did not contribute with any independent information when adjusting with known outcome predictors.
(iii) Radiologic intracranial hemorrhagic progression, an important predictor of long-term outcome, could not be predicted using MEA values.
(iv) MEA may in part be able to identify patients who arrive with TBI using COX inhibitors from a general coagulopathy.

Trauma-induced coagulopathy, one of the secondary injuries following TBI, is a poorly defined condition incorporating a hypo- and a hypercoagulable state (2). The pathophysiology is considered distinct from coagulopathy following other types of trauma, due to brain-specific features, e.g., high tissue factor levels, interaction between the disrupted blood–brain barrier/plasma proteins, and microparticle influence (35, 36). New methods to diagnose TIC have gained clinical interest, in particular platelet dysfunction, observed following TBI in both studies of animal models (37, 38) and human subjects without platelet inhibitory treatment (13, 38–41).

Platelet receptor values have but rarely (39) been characterized longitudinally following TBI. Numerous studies claim that trauma itself induces coagulopathy [reviewed in Ref. (35)]. Here, we saw that ~66% of the patients who had not received any COX inhibitor treatment exhibited pathologically low ASPI values upon admission, as defined by current reference levels. This finding is consistent with previous studies on coagulopathy prevalence in severe TBI (2). We show a longitudinal alteration of MEA values, with initially low values, followed by a later increase. This is consistent with a previous study (39), however, we measured over a longer time period and quantified the relationship longitudinally. The increase in MEA values was also observed in the absence of platelet transfusions, implying that this is a biological phenomenon reflecting a trauma-induced alteration of MEA values. This finding highlights the need for future studies to investigate MEA relations to clinically significant bleeding in TBI patients, even in the absence of platelet inhibitors. In addition, cutoff values and cutoffs related to elapsed time need to be established. In summary, this is the first quantitative examination of how MEA values alter following TBI.

In outcome analysis, CD plots of GOS and intracranial hemorrhagic progression exhibited a similar u-shaped relation between ASPI/GOS and ASPI/intracranial hemorrhagic progression, with both high and low levels suggesting worse outcomes. This implies that there might be ASPI values that are optimal to evade hemorrhagic progression and accordingly, affect GOS. However, these data should be interpreted cautiously, as the CD plots contained few observations at very low and high levels of ASPI. In univariate analysis (dependent variable GOS), both the first ASPI and TRAP values were significant. In multivariable analysis, this effect was no longer seen, meaning that ASPI and TRAP are meaningful for outcome prediction *per se*, but that they do not provide independent information when one has access to other base line variables. An interpretation of this is that pathological ASPI and TRAP values reflect a significant coagulopathy that could be related to deterioration and thus lower GCS. Prospective and clinician-blinded studies will be needed to confirm this. Previous studies show conflicting results. In one study, the TRAP, and ASPI receptors were independent predictors of mortality (39). In another study, unfortunately unadjusted for confounders, ADP and TRAP values were different between survivors and non-survivors in a mixed trauma population (13). Yet others have found ADP, but not ASPI, to be a predictor of mortality (41). Finally, there are also studies without relation between platelet function results and outcome, hemorrhagic progression, or mortality (42). The lack of congruency between these studies, including ours, indicates that there is a pressing need for future prospective studies on patients with severe TBI.

Radiologic intracranial hemorrhagic progression was a strong predictor of GOS. However, in univariate analysis of predictors for radiologic hemorrhagic progression, APTT at admission was the only significant variable. GCS and Stockholm CT score at admission were borderline significant, implying that a worse initial clinical status was associated with future progression and deterioration. The finding of APTT is interesting, but not the focus of this study.

Platelet inhibitors are common among TBI patients due to demographic alterations (5, 43). We found consistently lower ASPI values among patients on COX inhibitor treatment, congruent with others (39, 43). As has been similarly shown (43), the median ASPI was below the reference interval also for patients without known COX inhibitor treatment, making it difficult to distinguish pharmacologic from trauma-induced platelet receptor hypofunction. In outcome analysis COX inhibitor treatment was significantly related to GOS in univariate analysis, but not against radiologic intracranial hemorrhagic progression. In multivariable analysis, COX inhibitor treatment was no longer significant. COX inhibitor treatment has previously been shown to predict worsening lesions on follow-up radiology (7) and to predict unfavorable outcome [reviewed in Ref. (11)]. Other studies have failed to demonstrate this (42). As the majority of patients on COX inhibitor treatment are older, when adjusting for age, the true effect of the treatment might disappear. When we conducted the analysis omitting age adjustment, COX inhibitor treatment was a predictor of worse outcome, leading us to believe that not only could this account for previous discrepancies, but also, that until convincingly shown in a prospective randomized material, COX inhibitor treatment should be considered a risk factor for worse prognosis.

A theoretical treatment for platelet dysfunction is platelet transfusion. Based on ASPI levels, we distinguished an increased platelet function following transfusion, as expected (8). In outcome analysis, platelet transfusion was as a predictor of worse outcome in univariate, but not multivariable, analysis. These results should be interpreted very cautiously, since a group more heavily transfused often is one with more extensive injuries, and patients diagnosed with a hemorrhagic progression might have received transfusions to a larger extent than those who were not. Therefore, we cannot claim that transfusions are inadvisable. Yet, the implementation of MEA has resulted in diagnosis of presumptive pathology, for which there is no widely acknowledged treatment regimen (39). A review of five retrospective TBI studies where patients had antiplatelet therapy before admission found both beneficial and harmful effects of platelet transfusion therapy (44), whereas platelet transfusion was not significantly associated with outcome in later studies (45). In conclusion, the clinical value of platelet transfusions in the treatment of TIC therefore remains to be convincingly shown and especially in the setting of pathological MEA values in patients not receiving platelet inhibitors. Notably, the AABB (formerly the American Association of Blood Banks) could not recommend either for or against platelet transfusion for the subgroup of patients on antiplatelet therapy and traumatic intracranial hemorrhage (46).

This study should be pursued by a blinded randomized prospective multicenter study on isolated (non-multitrauma) TBI patients using GOS and lesion progression as outcome variables. MEA measurements should be taken at fixed, consecutive time points. Using these data, it would be possible to establish eligible reference intervals at different time points and conduct outcome analysis.

### Limitations

This study holds all the limitations of a retrospective study. Residual confounding, confounding by indication, and treatment bias must be assumed to exist in our data. These highlight problems with retrospective observational analyses and emphasize the need for blinded prospective trials. However, in lack of such MEA is often performed as a screening to identify if unconscious TBI patients are on platelet inhibitors. This will present the physician with a multitude of pathological MEA values in patients without platelet inhibitors, and without a metric for interpretation. This highly motivates an observational study such as this, despite its retrospective nature. Moreover, despite caveats, this is one of the first and larger studies to analyze a large cohort of mild-to-severe TBI patients and as we have had access to large amounts of prospectively collected data, we believe our data set is clinically valid for an NICU TBI population. Further, we have access to some unique variables like the trauma time, successfully registered in all prehospital records. Importantly, we have used this for a reliable characterization of platelet function longitudinally, which can rarely be done in equally large materials elsewhere. Still, MEA samples were to a large extent obtained at different time points, and treatments given at others. Moreover, our assessment of intracranial hemorrhage progression could be a bit sensitive as any lesion present was taken into account. Previous studies have defined set threshold, such as a minimal blood volume of 2 ml on the first CT scan (47). Further, we did not account for concomitant infection or disseminated intravascular coagulation (DIC) (48), both of which, but in particular DIC (48–50) could have confounded our data. This is complicated by the fact that it is difficult to distinguish between different types of coagulopathy following TBI (50). Altogether, we have been able to statistically compensate for some of the lack of standardization in our heterogeneous data set. Yet, the nature of our data and study design limits the extent to which conclusions can be drawn from this cohort, and our findings should at this point be seen as hypothesis generating. In aggregate, this study represents a large data set from a clinical setting that many physicians will be presented with and that will require decisions. This highly motivates this retrospective hypothesis generating study in wait of blinded prospective studies.

## Conclusion

We present the first larger investigation of the clinical utility of platelet function measurements using MEA in NICU treated TBI patients. Following TBI, a general longitudinal trend, with initially low MEA values and a subsequent increase over days is seen, indicating a pathophysiological link. MEA levels were affected by both COX inhibitor treatment and platelet transfusion. Progression of intracranial hemorrhage is an important predictor of poor TBI prognosis but MEA values could not significantly predict this condition. Both ASPI and TRAP values were associated with outcome, but did not add any independent information in presence of other outcome predictors. In summary, these findings warrant further prospective, blinded trials to full understand the utility of MEA in TBI patients.

## Ethics Statement

The study was carried out in accordance with Swedish legislation, the Declaration of Helsinki, and the specific recommendations stipulated by the local ethics committee in Stockholm County, Centrala Etikprövningsnämnden (the Central Ethical Review Board). The study was exempt from written informed consent, as it was carried out retrospectively on data base material, was purely observational, and did not inflict on patient treatment. The local ethics committee (the Central Ethical Review Board) approved the protocol of this study (diary numbers 2014/1488-31/5 and 2015/1675-31/1).

## Author Contributions

CL, ET, AF, MN, DN, B-MB, and MS designed and planned the study. CL, ET, and MN acquisitioned the data. CL, ET, AF, and DN analyzed and interpreted the data. CL drafted the manuscript. CL, ET, MN, AF, DN, MS, and B-MB revised the manuscript critically. All the authors read and approved the final manuscript and agreed to be accountable for all aspects of the work.

## Conflict of Interest Statement

None of the coauthors have received any payment from a third party for any aspect of the submitted work. The authors have no financial relationships to disclose. B-MB has participated and lectured during Roche “user meetings,” which is an annual gathering of researchers and clinicians to share experiences and research results following usage of Multiplate^®^. CL has participated in one of those meetings. During the meeting, which each time extended for 1 day, food and beverages were provided free of charge. None of the other coauthors have any conflicts of interest to declare.
