# Supplementary material for: Assessment of Platelet Function in Traumatic Brain Injury—A Retrospective Observational Study in the Neuro-Critical Care Setting
Source: Front Neurol. 2018 Jan 26;9:15. doi: 10.3389/fneur.2018.00015 (PMC5790800; doi:10.3389/fneur.2018.00015)
Supplement: Supplementary file 1 [file presentation_1.PDF]

Supplementary material

Clinical Value of Platelet Function Monitoring following Traumatic Brain Injury

Caroline Lindblad, Eric Peter Thelin, Michael Nekludov, Arvid Frostell, David W Nelson, Mikael Svensson, and Bo-Michael Bellander*.

*= Corresponding author

Correspondence:
Dr Bo-Michael Bellander
bo-michael.bellander@ki.se

Supplementary Materials and Methods

Statistical analysis

The statistical software program Rstudio® (R Foundation for Statistical Computing, Vienna, Austria; http://www.R-project.org) was used in all calculations.

Demographics

Continuous variables are described with mean ± standard deviation (SD) if normally distributed or otherwise with median (interquartile range). Categorical variables are described with counts and percentages.

Correlation between different platelet receptors

We (1) and others (2) have previously shown ASPI to be the most promising platelet receptor to study. However, one might hypothesize a correlation and possible covariance between different platelet receptor values. This was also shown in a previous study (2). This pattern might make it inadvisable to use all of the MEA receptors at once, since it would add a neglectable amount of information, and occasionally could be directly misleading as their internal covariation might lead to a positive over-interpretation of the results obtained from them. Agreement between them, however, should not be examined, since they cannot from a biological point of view, be used interchangeably. We examined correlation between MEA receptors using scatterplots, and by calculating the Spearman correlation (ρ) between the first MEA values. This guided us to pursue our investigations on one of the MEA receptors.

Pharmacologic modulation of MEA

We compared the first MEA ASPI measurements between patients on COX inhibitors and patients without COX inhibitors. The rational for this is that COX inhibitors are the pathway specific inhibitors of the ASPI response (3). COX inhibitors were defined as either acetylsalicylic compounds or non-steroidal anti-inflammatory drugs. The doses of these substances and the indications for them varied between patients. A patient was considered to be on the medication if having taken it regularly during the time preceding hospital admission. A patient was also considered to have the therapy if it was so noted in the hospital medical record. Naturally, it is difficult to estimate compliance using this strategy. Indications for COX inhibitor treatment are commonly cardiovascular diseases (acetylsalicylic compounds) or pain conditions (non-steroidal anti-inflammatory drugs) in Sweden. The specific indication for each patient was beyond the scope of the present manuscript. For analysis, we used a subset of the original data set, defined as patients on which the first MEA sample had been obtained before the first platelet transfusion (or, if no platelet transfusion was undertaken, the first MEA value). This criteria was used, since we otherwise would have risked including patients who had received platelet transfusions, which could have confounded or analysis of COX inhibitor impact on MEA values. We included only the first measurement, as that measurement probably reflected the least treatment bias and because COX inhibitor treatment is halted following admittance to the NICU.

In order to examine whether the MEA value was altered following platelet transfusion, we compared the first ASPI measurement with the second dito. Patients included had not obtained any platelet transfusions before the first MEA sample, but obtained a platelet transfusion before the second MEA sample. We did not account for how long time that had passed between the transfusion and the second MEA measurement. For these analyses, the distribution of the variable ASPI was examined using the Shapiro Wilk test, and inferential analysis were

conducted using the Mann Whitney U test (COX inhibitors) and the Wilcoxon signed rank test (platelet transfusion).

Longitudinal analyses

We assessed longitudinal changes of MEA values. ”Time from trauma” was defined as the difference between the MEA measurement time and the trauma time as noted in the pre-hospital records. Analysis was conducted using linear mixed effect models in the lme4 (4) and lmerTest (5) packages in R. ASPI was used as dependent variable. As fixed effects, we used time from trauma, platelet count, and the dichotomous variable COX inhibitor treatment. As random effects, we used random intercepts for subject id. Model criteria were evaluated graphically and deemed to be fulfilled. We compared nested models using likelihood ratio tests. The compiled model with the lowest Akaike Information Criterion (AIC) value was chosen. P-values are reported as the p-values from the likelihood ratio tests or as otherwise stipulated.

The importance of MEA measurements for patient outcome

Missing values of the data set relevant for outcome analysis were plotted (Figure S1) using a modified code from the neato package (6). Several variables in the plot contained missing values to a large extent. To account for this, we employed a multiple imputation approach. We made seven imputations of the dataset using the R package mice (7). The individual imputations and the pooled result from them, were used in subsequent analyses. This approach has been used by the International Mission for Prognosis and Analysis of Clinical Trials in TBI (IMPACT) study group (8) and is favored by the statistical literature (9). To visualize the importance of the first ASPI value on outcome prediction compared with other variables, a decision tree was constructed from each imputation. The dependent variable was GOS (10). Independent variables were those previously shown to be the major predictors for TBI outcome (IMPACT variables) (11), combined with coagulation tests clinically available at our clinic. Calculations were made using recursive partitioning in the R package rpart (12) and rattle (13).

Finally, we made two proportional odds regressions, one using GOS and one using radiologic intracranial hemorrhagic progression as dependent variables. In the model using GOS, univariate analysis was made on the original data (not imputed). Variables emanating significant were then analyzed one-by-one together with the base model (the IMPACT variables) versus the base model alone in likelihood ratio tests (14). This step-up model was done on each of the 7 imputations of the data set and is presented as the mean p value, mean Nagelkerke pseudo-R^2, and the ΔR^2 between the base model and the extended model. Next, we undertook a step-down approach, incorporating all variables significant in univariate analysis into a multivariable model and then successively omitted the variable with the highest p value, until all values present in the model had a significant p value. This was done on the pooled imputations. For the regressions on radiologic intracranial hemorrhagic progression, no base model similar to IMPACT exists, why we limited our analysis to univariate analysis followed by a step-down model, on the pooled imputed data. Variables included in univariate analysis were chosen with guidance from Fabbri and colleagues (15), together with variables hypothesized to modulate platelet function or coagulation. The proportional odds regression model was made on all seven imputations of the data using the R package rms (14). The results of the proportional odds regression models are reported as the Odds ratio (OR) and confidence interval (CI) for one imputation. We also report the pooled p-values for all imputations. The Nagelkerke’s pseudo-R^2 are reported as the mean for all the imputations.

Supplementary References

1. Nekludov M, Bellander BM, Blomback M, Wallen HN. Platelet dysfunction in patients with severe traumatic brain injury. J Neurotrauma. 2007;24(11):1699-706.
2. Kutcher ME, Redick BJ, McCreery RC, Crane IM, Greenberg MD, Cachola LM, et al. Characterization of platelet dysfunction after trauma. J Trauma Acute Care Surg. 2012;73(1):13-9.
3. Beynon C, Scherer M, Jakobs M, Jung C, Sakowitz OW, Unterberg AW. Initial experiences with Multiplate(R) for rapid assessment of antiplatelet agent activity in neurosurgical emergencies. Clin Neurol Neurosurg. 2013;115(10):2003-8.
4. Bates D, Machler M, Bolker BM, Walker SC. Fitting Linear Mixed-Effects Models Using lme4. Journal of Statistical Software. 2015;67(1):1-48.
5. Kuznetsova A, Brockhoff PB, Haubo Bojesen Christensen R. lmerTest: Tests in Linear Mixed Effects Models. . R package version 2.0-33.2016.
6. Tierney N. neato: Various Useful Functions That I Repeatedly Use. R package version 0.0.2.900 ed2015.
7. van Buuren S, Groothuis-Oudshoorn K. mice: Multivariate Imputation by Chained Equations in R. Journal of Statistical Software. 2011;45(3):1-67.
8. Murray GD, Butcher I, McHugh GS, Lu J, Mushkudiani NA, Maas AI, et al. Multivariable prognostic analysis in traumatic brain injury: results from the IMPACT study. J Neurotrauma. 2007;24(2):329-37.
9. Marshall A, Altman DG, Royston P, Holder RL. Comparison of techniques for handling missing covariate data within prognostic modelling studies: a simulation study. BMC Med Res Methodol. 2010;10:7.
10. Jennett B, Bond M. Assessment of outcome after severe brain damage. Lancet. 1975;1(7905):480-4.
11. Marmarou A, Lu J, Butcher I, McHugh GS, Mushkudiani NA, Murray GD, et al. IMPACT database of traumatic brain injury: design and description. J Neurotrauma. 2007;24(2):239-50.
12. Therneau T, Atkinson B, Ripley B. rpart: Recursive Partitioning and Regression Trees. 2015.
13. Williams Graham J. Data Mining with Rattle and R: The art of excavating data for knowledge discovery: Springer; 2011.
14. Harrell JFE. rms: Regression Modeling Strategies. 2016.
15. Fabbri A, Servadei F, Marchesini G, Bronzoni C, Montesi D, Arietta L, et al. Antiplatelet therapy and the outcome of subjects with intracranial injury: the Italian SIMEU study. Crit Care. 2013;17(2):R53.

Supplementary Figures and Figure Legends

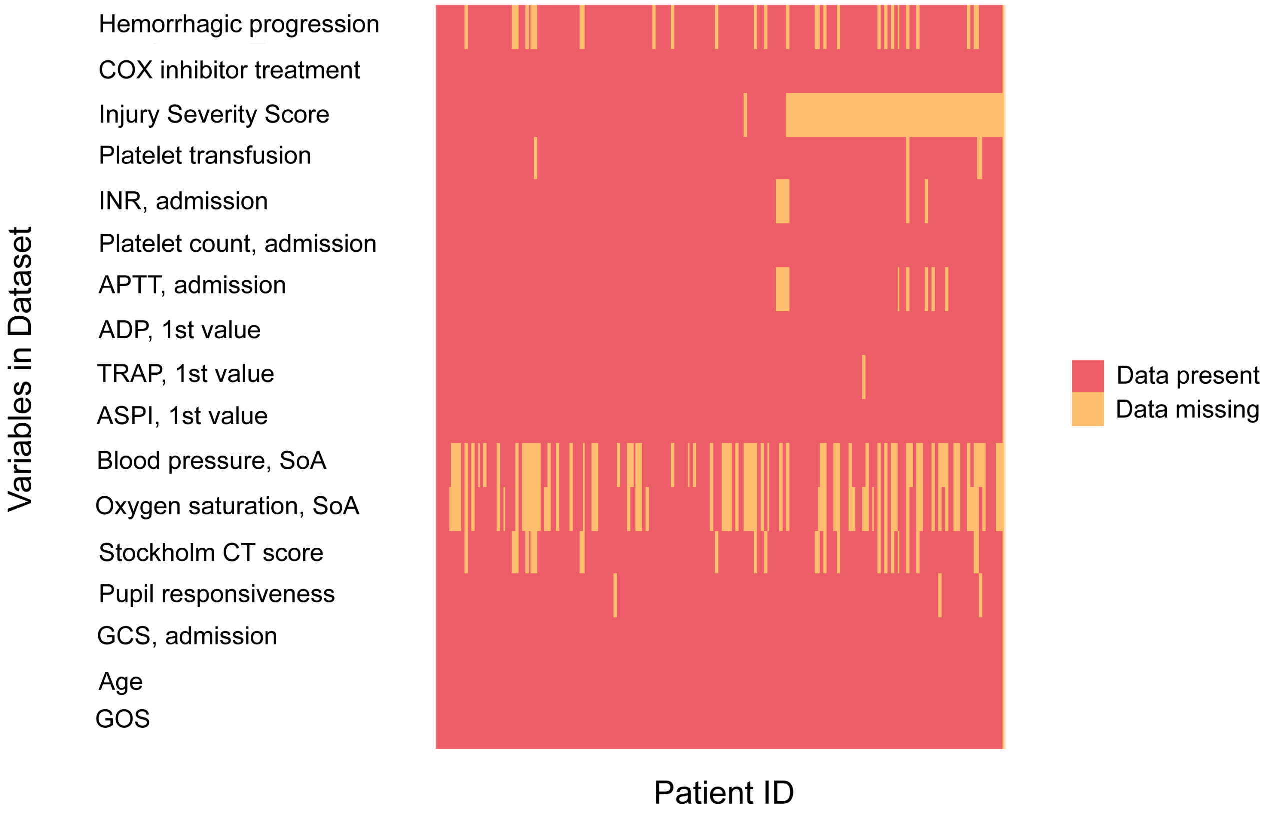

Supplementary Figure 1 (S1). Missing map of all variables relevant for outcome analysis.

Supplementary Figure 1 (S1). Variables of importance for outcome analysis were plotted in order to visualize the amount of missing data in each variable. MEA analysis was an inclusion criteria. Abbreviations: ADP, $P2Y_{12}$ receptor; APTT, activated partial thromboplastin time; ASPI, arachidonic acid receptor; BP, blood pressure; COX, cyclooxygenase; CT, computerized tomography; GCS, Glasgow Coma Scale; GOS, Glasgow Outcome Scale; INR, international normalized ratio; MEA, multiple electrode aggregometry; SoA, Scene of Accident; TRAP, thrombin receptor.

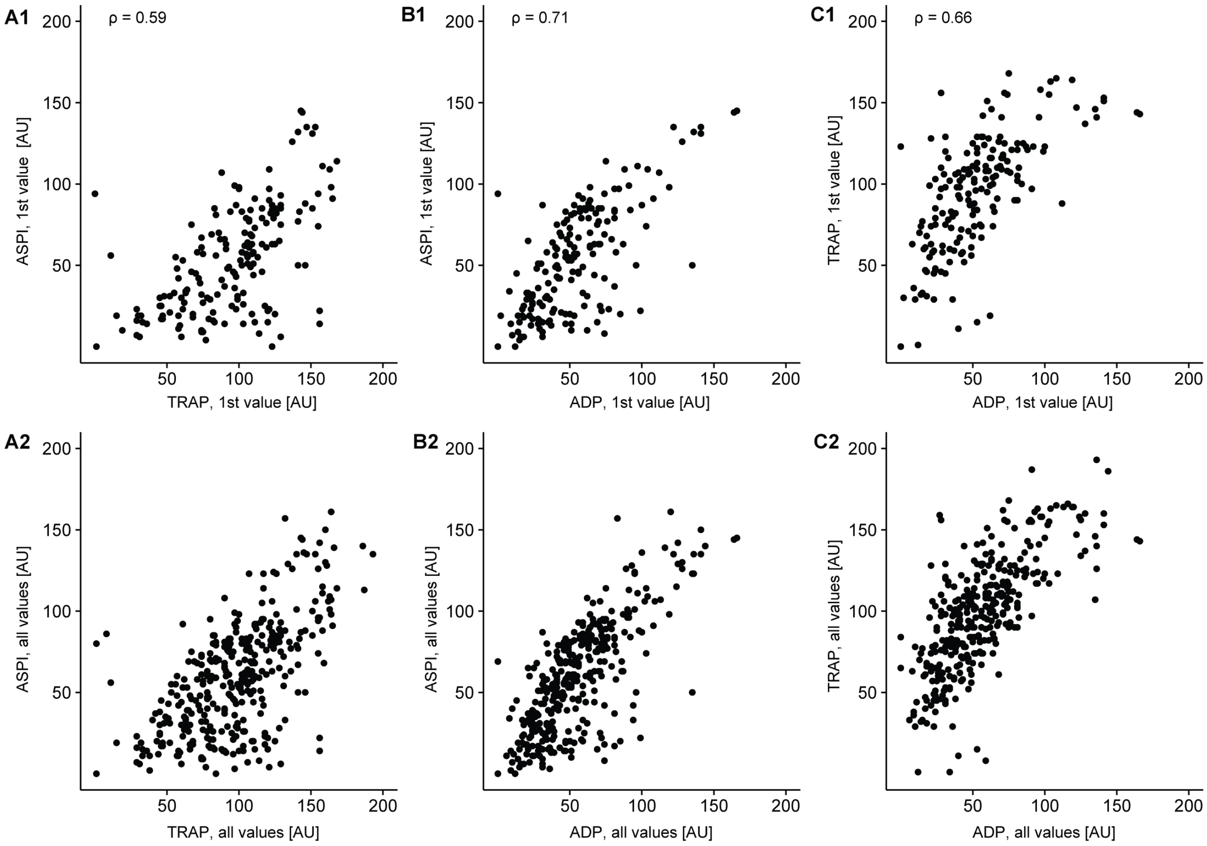

Supplementary Figure 2 (S2). Correlations between different platelet receptor values
Supplementary Figure 2 (S2). The first MEA values for the three different receptors measured with MEA were positively correlated with one another **(A1-C1)**. All measurements at all time points are also demonstrated **(A2-C2)**. Abbreviations: ASPI, arachidonic acid receptor; ADP, $P2Y_{12}$ receptor; MEA, multiple electrode aggregometry; TRAP, thrombin receptor.

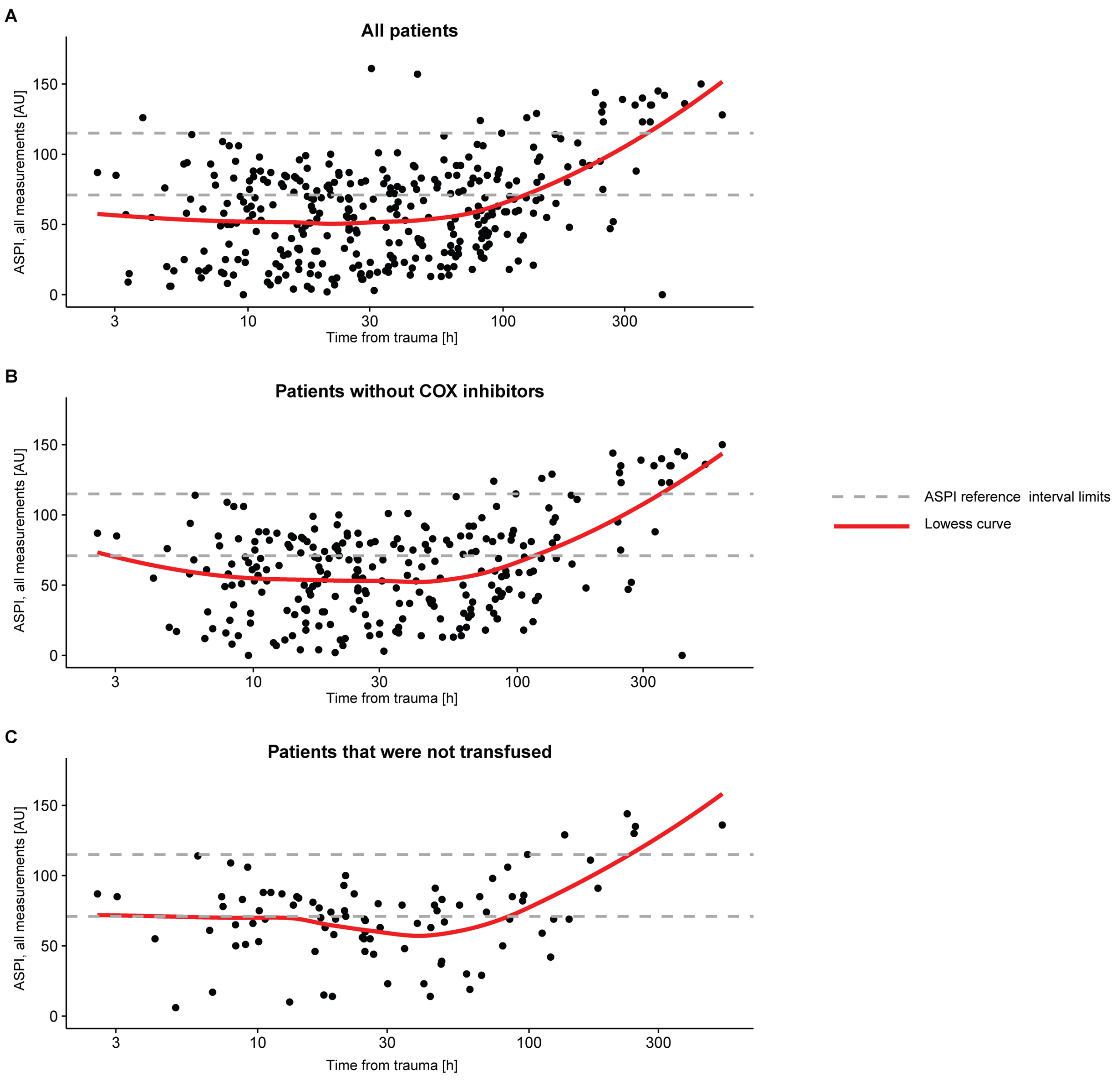

Supplementary Figure 3 (S3). Platelet function is altered over time following TBI
Supplementary Figure 3 (S3). All patients across all time points are depicted in **(A)**. As each individual might have undergone multiple measurements, each data point represents one measurement and not one individual. There was a trend for an initial low value in arachidonic acid receptor (ASPI) values, followed by a period of relatively constant values and subsequently an increase in ASPI, as time increased. In **(B)**, we demonstrate how this trend was similar when comparing patients without cyclooxygenase (COX) inhibitor treatment prior to admission. In **(C)**, we show that this trend was stable even when excluding patients who were not transfused during their stay at the neurointensive care unit (NICU). The dark gray dashed line denotes the ASPI reference interval (71-115 AU), and the red line the trend line (locally weighted scatterplot smoother [lowess]). Taken together, this argues in favor of a temporal variation of platelet function following a trauma. Abbreviations: AU, area under the curve.

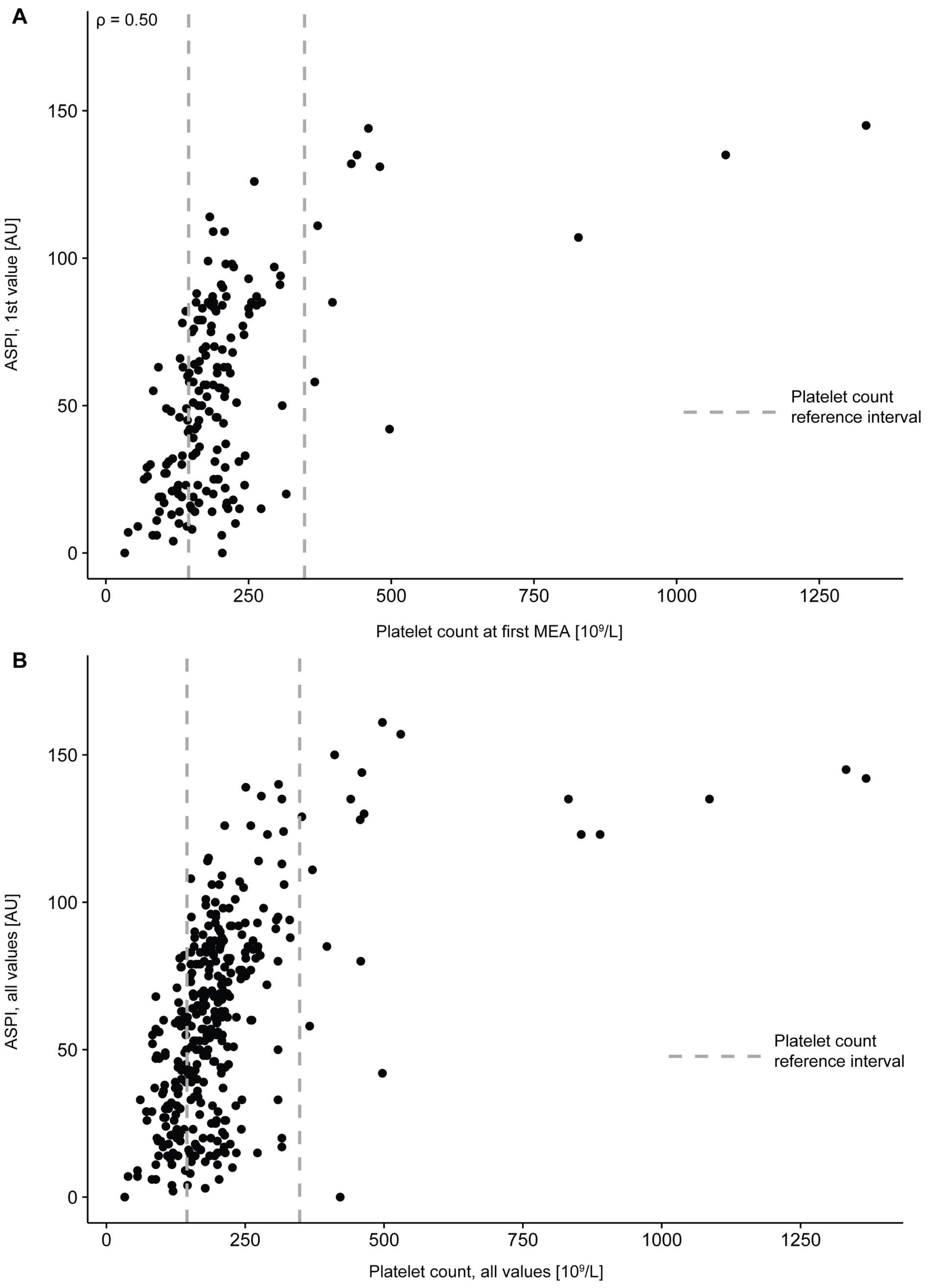

Supplementary Figure 4 (S4). MEA is correlated with platelet count

Supplementary Figure 4 (S4). Platelet count was shown to be positively, linearly correlated with arachidonic acid receptor (ASPI) values. The dark gray dashed line denotes the platelet count reference interval (145 - 348 [10^9/L]). This implies that there was an association between platelet count and MEA values, that should be accounted for.

Supplementary Tables and Table Legends

Supplementary Table 1 (Table S1). Step up model of variables predicting Final GOS

**Independent variable**	**Mean p**	**Nagelkerke pseudo-R^2**	**$\Delta R^2 = R^2$ Base – R^2 Base + Variable**
IMPACT	-	0.372	-
IMPACT + ASPI, 1st value	0.381	0.376	0.00307
IMPACT +TRAP, 1st value	0.164	0.380	0.00781
IMPACT + APTT, admission	0.0613	0.388	0.0157
IMPACT + Platelet transfusion	0.0267	0.392	0.0197
IMPACT + COX inhibitor treatment	0.184	0.380	0.00770
IMPACT + Radiologic progression intracranial hematoma	0.005	0.406	0.0338

Supplementary Table 1 (Table S1). Step up model of variables predicting Final GOS
Supplementary table 1 (Table S1). Variables significant in univariate analysis were compared individually with "a base model" using likelihood ratio tests. Base were considered to be the IMPACT variables (11) age, GCS on admission, pupil responsiveness, Stockholm CT score, Saturation at SoA, Blood pressure at SoA. Abbreviations: APTT, activated partial thromplastin time; ASPI, arachidonic acid receptor; COX, cyclooxygenase; CT, computerized tomography; GCS, Glasgow Coma Scale; SoA, scene of accident; TRAP, thrombin receptor.

Supplementary Table 2 (S2). Univariate analysis of variables and correlations to radiologic intracranial hemorrhagic progression

Independent variable	P value	Pseudo R^2
Sex	0.85	NS
Age	0.37	NS
GCS at admission	0.057	0.033
Stockholm CT Score	0.064	0.031
Injury Severity Score	0.9980	NS
ASPI, 1st value	0.1258	NS
ADP, 1st value	0.8952	NS
TRAP, 1st value	0.2152	NS
Platelet count at admission	0.1699	NS
INR at admission	0.2784	NS
APTT at admission	0.0178	0.058
COX inhibitor treatment	0.27	NS

Supplementary Table 2 (S2). Univariate analysis of variables predicting radiologic intracranial hemorrhagic progression

Supplementary Table 2 (S2). Results from univariate analysis of independent variables assumed to affect radiologically verified intracranial hemorrhagic progression are depicted. Apart from variables that had previously been used by Fabbri et al (15), variables hypothesized to modulate platelet function or coagulation were included. Platelet transfusion and COX inhibitor treatment were used as dichotomous variables. Abbreviations: ADP, $P2Y_{12}$ receptor; APTT, activated partial thromplastin time; ASPI, arachidonic acid receptor; COX, cyclooxygenase; CT, computerized tomography; GCS, Glasgow Coma Scale; INR, International Normalized ratio; SoA, scene of accident; TRAP, thrombin receptor.
